# Supplementary material for: MMETHANE: interpretable AI for predicting host status from microbial composition and metabolomics data
Source: Microbiome. 2025 Dec 8;14:21. doi: 10.1186/s40168-025-02270-z (PMC12802134; doi:10.1186/s40168-025-02270-z)
Supplement: Supplementary file 2 — Supplementary Material 1: Supplementary Figure S1. The PubChem CACTVS fingerprint yielded the most robust predictive performance when used as a molecular similarity measure for MMETHANE. Five-fold cross-validated AUC scores for prediction of host status on the six datasets in the compendium are shown, using five different molecular similarity measures (PubChem CACTVS, Morgan, MAP4, MQN and InfoMax). Box plots indicate medians and 95% intervals for runs over ten random seeds. Yellow diamonds indicate the top score or scores (if multiple scores were not significantly different from the top score, p > 0.05). Supplementary Table S1. Compendium of paired microbial composition and metabolomics datasets. NMR = nuclear magnetic resonance. CDI = Clostridioides difficile infection. HPLC–MS/MS = high-performance liquid chromatography-tandem mass spectrometry. IBD = inflammatory bowel disease. LC–MS = liquid chromatography-mass spectrometry. ESRD = End-stage renal disease. Supplementary Table S2. Chemical fingerprints assessed and rationale for selection. Supplementary Table S3. Benchmarking results for comparator methods using aggregated taxa or metabolites based on pre-defined groupings as inputs. Benchmarking methods were evaluated on aggregated taxonomic family or metabolic class-level predictors. Cross-validated AUC values with ranges are reported as described in the main text and Methods. Results from aggregated predictors and non-aggregated predictors for each dataset and each benchmark method were compared with Mann–Whitney U tests, with bolded scores indicating significantly better results. Overall, models given non-aggregated data performed better than models given aggregated data. Supplementary Table S4. Modality (metabolite or taxa) of predictors found when both metabolomics and taxa abundance data were given to models as inputs. LR = lasso logistic regression, RF = random forests, FFNN = feedforward neural networks. For LR, features were retained if their 95% cross [file 40168_2025_2270_MOESM1_ESM.docx]

Supplementary Figures and Tables


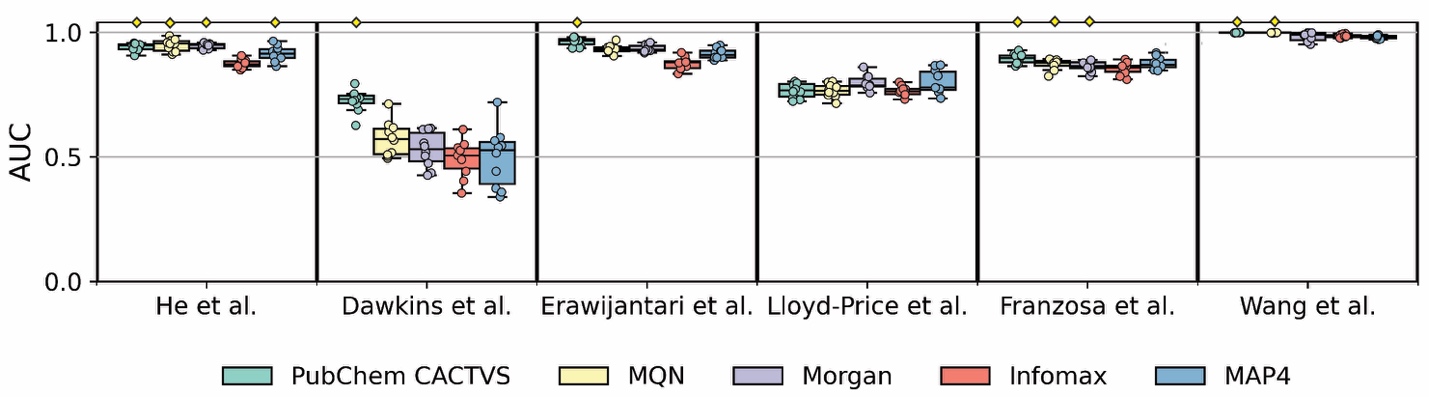


Supplementary Figure 1. The PubChem CACTVS fingerprint yielded the most robust predictive performance when used as a molecular similarity measure for MMETHANE. Five-fold cross-validated AUC scores for prediction of host status on the six datasets in the compendium are shown, using five different molecular similarity measures (PubChem CACTVS, Morgan, MAP4, MQN and InfoMax). Box plots indicate medians and 95% intervals for runs over ten random seeds. Yellow diamonds indicate the top score or scores (if multiple scores were not significantly different from the top score, *p* > 0.05).

Supplementary Table 1. Compendium of paired microbial composition and metabolomics datasets. NMR = nuclear magnetic resonance. CDI = *Clostridioides difficile* infection. HPLC-MS/MS = high-performance liquid chromatography-tandem mass spectrometry. IBD = inflammatory bowel disease. LC-MS = liquid chromatography-mass spectrometry. ESRD = End-stage renal disease.

| Dataset | Host status | # ID’d metabolites | Metabolomics method | Microbial sequencing method |
| --- | --- | --- | --- | --- |
| He et al | 26 breast fed  16 formula fed | 108 | H1-NMR | 16S rRNA |
| Dawkins et al | 14 CDI recurrers  33 non-recurrers | 1005 | HPLC-MS/MS, ID’d and annotated by Metabolon | 16S rRNA |
| Erawijantari et al | 42 w/ previous gastrectomy  54 controls | 511 | capillary electrophoresis time-of-flight | metagenomics |
| Lloyd-Price et al | 79 IBD  26 controls | 290 | 4 LC-MS analyses: (1) HILIC-pos, (2) HILIC-neg, (3) C18 neg, (4) C8 pos lipids | metagenomics |
| Franzosa et al | 121 IBD  34 controls | 232 | 4 LC-MS analyses: (1) pos ion polar, (2) neg ion polar, (3) neg ion intermediate polar, (4) lipids (polar & non-polar) | metagenomics |
| Wang et al | 220 ESRD  67 controls | 156 | HPLC-MS/MS, ID'ed and annotated from in-house database | metagenomics |

Supplementary Table 2. Chemical fingerprints assessed and rationale for selection.

| **Type** | **Fingerprint** | **# Dimensions** | **Numerical type** | **Reasoning** |
| --- | --- | --- | --- | --- |
| Substructure keys | PubChem CACTVS [[27](#_ENREF_27)] | 880 | Binary | Most comprehensive of substructure-keys |
|  | Molecular quantum numbers (MQN) [[29](#_ENREF_29)] | 42 | Counts | Least complex of substructure-keys |
| Topological | Morgan  (Extended connectivity fingerprint) [[57](#_ENREF_57)] | 1024 | Binary | Commonly used as a comparison in other publications |
| Substructure + Topological | MAP4 (Substructure + Atom Pair) [[59](#_ENREF_59)] | 1024 | Counts | Developed specifically for untargeted metabolomics |
| Deep learning | Infomax [[30](#_ENREF_30), [60](#_ENREF_60)] | 300 | Continuous $\in$(0,1) | Highest performance in a comparative study |

**Supplementary Table 3. Benchmarking results for comparator methods using aggregated taxa or metabolites based on pre-defined groupings as inputs.** Benchmarking methods were evaluated on aggregated taxonomic family or metabolic class-level predictors. Cross-validated AUC values with ranges are reported as described in the main text and Methods. Results from aggregated predictors and non-aggregated predictors for each dataset and each benchmark method were compared with Mann-Whitney U tests, with bolded scores indicating significantly better results. Overall, models given non-aggregated data performed better than models given aggregated data.

|  |  | **AdaBoost** | **Lasso LR** | **RF** | **FFN** |
| --- | --- | --- | --- | --- | --- |
| Dawins et al. | Group Families & Classes | 0.395 [0.395, 0.408] | 0.382 [0.382, 0.382] | 0.44 [0.422, 0.442] | 0.81 [0.752, 0.83] |
|  | No Grouping | **0.676 [0.676, 0.706]** | **0.694 [0.694, 0.694]** | **0.571 [0.549, 0.584]** | 0.756 [0.737, 0.766] |
| Erawijantari et al. | Group Families & Classes | 0.863 [0.849, 0.874] | 0.812 [0.808, 0.833] | 0.89 [0.88, 0.892] | 0.88 [0.871, 0.886] |
|  | No Grouping | 0.862 [0.858, 0.876] | **0.906 [0.902, 0.918]** | 0.886 [0.876, 0.889] | **0.902 [0.89, 0.912]** |
| Franzosa et al. | Group Families & Classes | 0.733 [0.725, 0.742] | 0.758 [0.728, 0.771] | 0.746 [0.725, 0.753] | 0.884 [0.872, 0.893] |
|  | No Grouping | 0.766 [0.726, 0.802] | **0.827 [0.814, 0.833]** | 0.739 [0.731, 0.741] | **0.901 [0.892, 0.914]** |
| He et al. | Group Families & Classes | 0.834 [0.817, 0.854] | 0.794 [0.752, 0.81] | 0.834 [0.798, 0.856] | 0.942 [0.931, 0.951] |
|  | No Grouping | **0.93 [0.93, 0.95]** | **0.908 [0.874, 0.918]** | **0.934 [0.918, 0.95]** | **0.807 [0.768, 0.821]** |
| Lloyd-Price et al. | Group Families & Classes | 0.528 [0.506, 0.542] | 0.638 [0.576, 0.655] | 0.571 [0.548, 0.582] | 0.677 [0.638, 0.694] |
|  | No Grouping | **0.671 [0.66, 0.714]** | **0.701 [0.694, 0.71]** | **0.628 [0.598, 0.63]** | 0.681 [0.655, 0.704] |
| Wang et al. | Group Families & Classes | 0.907 [0.892, 0.912] | 0.917 [0.909, 0.921] | 0.857 [0.843, 0.864] | 0.95 [0.943, 0.952] |
|  | No Grouping | **0.99 [0.986, 0.991]** | **0.994 [0.99, 0.995]** | **0.935 [0.926, 0.944]** | **0.983 [0.978, 0.985]** |

Supplementary Table 4. Modality (metabolite or taxa) of predictors found when both metabolomics and taxa abundance data were given to models as inputs. LR = lasso logistic regression, RF = random forests, FFNN = feedforward neural networks. For LR, features were retained if their 95% cross-validated odds interval over 10 seeds did not contain 0. For RFs and AdaBoost, features were retained if their 95% cross-validated Gini importance over 10 seeds did not contain 0. For FNNs, the top 10 features were retained, ranked based on the mean of the integrated gradient of each feature over all subjects and 10 seeds.

|  |  | **MMETHANE** | **LR** | **RF** | **AdaBoost** | **FFNN** |
| --- | --- | --- | --- | --- | --- | --- |
| He et al | # Metabolite predictors | 1 | 3 | 2 | 1 | 10 |
|  | # Taxa predictors | 0 | 2 | 0 | 0 | 0 |
| Dawkins et al | # Metabolite predictors | 11 | 7 | 4 | 1 | 10 |
|  | # Taxa predictors | 0 | 0 | 0 | 0 | 0 |
| Erawijantari et al | # Metabolite predictors | 3 | 4 | 7 | 1 | 10 |
|  | # Taxa predictors | 16 | 1 | 2 | 0 | 0 |
| Lloyd-Price et al | # Metabolite predictors | 4 | 4 | 3 | 1 | 9 |
|  | # Taxa predictors | 21 | 0 | 0 | 0 | 1 |
| Franzosa et al | # Metabolite predictors | 2 | 4 | 9 | 2 | 10 |
|  | # Taxa predictors | 35 | 1 | 1 | 0 | 0 |
| Wang et al | # Metabolite predictors | 6 | 8 | 11 | 7 | 7 |
|  | # Taxa predictors | 18 | 4 | 2 | 0 | 3 |

**Supplementary Table 5. Embedding dimensions for taxa and metabolites on the datasets in the compendium.** Note that the embedding dimensions for metabolites and taxa were approximately the same within and across datasets, with the exception of He et al. and Dawkins et al., which had lower embedding dimensions for taxa, because these datasets used 16S rRNA phylotyping in contrast to metagenomic sequencing used in the other datasets.

|  | **Metabolite embedding dimensions** | **Taxa embedding dimensions** |
| --- | --- | --- |
| He et al. | 24 [17, 28] | 6 [0, 10] |
| Lloyd-Price et al. | 26 [21, 30] | 26 [3, 30] |
| Franzosa et al. | 24 [19, 29] | 28 [2, 30] |
| Wang et al. | 27 [23, 30] | 27 [2, 30] |
| Erawijantari et al. | 26 [22, 30] | 28 [2, 30] |
| Dawkins et al. | 28 [23, 30] | 6 [2, 10] |

**Supplementary Table 6. Benchmarking results for MMETHANE using different embedding methods for detectors.** We compared MMETHANE’s performance on the data compendium using UMAP, PCoA, and tSNE for embeddings of metabolites and taxa. In three of the six datasets, there was no statistically significant difference between results. Cross-validated AUC values with ranges are reported as described in the main text and Methods. The Kruskal-Wallis test was used to determine any significant differences among results with different embedding methods. For the datasets in which results were statistically significant, the Mann-Whitney U test was used further differentiate between embedding methods. Bolded scores indicating significantly better results. In three of the datasets, there were no significant differences between the embedding methods. In Wang et al. and Dawkins et al., PCoA performed significantly better than UMAP and tSNE, while in Erawijantari et al., PCoA and tSNE performed significantly better than UMAP.

|  | **UMAP** | **PCoA** | **tSNE** |
| --- | --- | --- | --- |
| He et al. | 0.94 [0.926, 0.963] | 0.945 [0.94, 0.946] | 0.93 [0.917, 0.936] |
| Lloyd-Price et al. | 0.653 [0.598, 0.688] | 0.687 [0.66, 0.7] | 0.681 [0.66, 0.697] |
| Franzosa et al. | 0.888 [0.869, 0.902] | 0.906 [0.896, 0.912] | 0.89 [0.88, 0.894] |
| Wang et al. | 0.98 [0.971, 0.986] | **0.997 [0.995, 0.998]** | 0.978 [0.97, 0.982] |
| Erawijantari et al. | 0.939 [0.932, 0.942] | **0.956 [0.949, 0.962]** | **0.951 [0.948, 0.956]** |
| Dawkins et al. | 0.539 [0.52, 0.557] | **0.675 [0.663, 0.693]** | 0.367 [0.326, 0.388] |

**Supplementary Table 7. Median run time per seed in minutes on an Apple M2 Max 12 core CPU.**

|  | **# input metabolites** | **# input taxa** | **# samples** | **Median [5%, 95%] run time per seed (in minutes) over 10 seeds** |
| --- | --- | --- | --- | --- |
| He et al. | 98 | 349 | 42 | 2.0 [0.9, 4.7] |
| Lloyd-Price et al. | 287 | 120 | 88 | 1.7 [1.2, 3.3] |
| Franzosa et al. | 232 | 207 | 155 | 1.6 [1.2, 2.9] |
| Wang et al. | 156 | 197 | 287 | 1.4 [1.2, 1.5] |
| Erawijantari et al. | 264 | 217 | 96 | 3.0 [1.1, 5.6] |
| Dawkins et al. | 417 | 101 | 46 | 3.2 [2.0, 5.6] |

**Supplementary Table 8. Feedforward Neural Network (FFNN) layer sizes for each dataset in the compendium. Note that the input layer size reflects the total number of taxa and metabolites in the dataset.**

|  | **input** | **hidden layer 1** | **hidden layer 2** | **hidden layer 3** | **output** |
| --- | --- | --- | --- | --- | --- |
| He et al | 447 | 61 | 36 | 36 | 1 |
| Lloyd-Price et al | 407 | 58 | 34 | 34 | 1 |
| Franzosa et al | 439 | 60 | 36 | 36 | 1 |
| Wang et al | 353 | 54 | 32 | 32 | 1 |
| Erawijantari et al | 481 | 63 | 37 | 37 | 1 |
| Dawkins et al | 518 | 65 | 39 | 39 | 1 |
